# Timed hazard networks: Incorporating temporal difference for oncogenetic analysis

**DOI:** 10.1371/journal.pone.0283004

**Published:** 2023-03-16

**Authors:** Jian Chen

**Affiliations:** Department of Computer Science and Engineering, University at Buffalo, Buffalo, NY, United States of America; Vinnytsia National Technical University, UKRAINE

## Abstract

Oncogenetic graphical models are crucial for understanding cancer progression by analyzing the accumulation of genetic events. These models are used to identify statistical dependencies and temporal order of genetic events, which helps design targeted therapies. However, existing algorithms do not account for temporal differences between samples in oncogenetic analysis. This paper introduces Timed Hazard Networks (TimedHN), a new statistical model that uses temporal differences to improve accuracy and reliability. TimedHN models the accumulation process as a continuous-time Markov chain and includes an efficient gradient computation algorithm for optimization. Our simulation experiments demonstrate that TimedHN outperforms current state-of-the-art graph reconstruction methods. We also compare TimedHN with existing methods on a luminal breast cancer dataset, highlighting its potential utility. The Matlab implementation and data are available at https://github.com/puar-playground/TimedHN

## Introduction

The progression of human cancer can be understood as an evolutionary process at the cellular level [1]. This process involves accumulating genetic changes, including mutations, copy number alterations, and modifications in DNA methylation and gene expression, which provide cancer cells with selective advantages and result in clonal expansion [2]. The accumulations of genetic alterations often exhibit a consistent pattern in different patients, for example, the sequential accumulation of APC→K-RAS→TP53 gene mutations in colorectal carcinogenesis [3]. However, identifying complex dependencies among a larger number of genetic alterations remains an open question with important implications for patient treatment.

During the past 20 years, a dozen oncogenetic modeling methods have been developed for cross-sectional samples. Assuming different individuals’ genetic alteration profiles are independent observations from the same multivariate stochastic process, these methods construct directed graphical models that reflect the dependencies or causalities between genetic alterations among the patient population using cross-sectional samples. Specifically, each node stands for a genetic event whose probability depends on the events connected by incoming edges. Commonly used oncogenetic models infer three types of graphs. The first type of models infer a tree or forest structure where a single event may have multiple outgoing edges but have at most one incoming edge (e.g., oncotrees [4], METREX [5], Mtreemix [6], CAPRESE [7]). This structure is used for simplicity and is expected to have a lower false-positive rate because it only captures the dominant factors in oncogenesis. The second class of methods tends to adopt structural learning for bayesian networks to learn a directed acyclic graph (DAG) (e.g., Conjunctive Bayesian Networks [8], DiProg [9], Bayesian Mutation Landscape [10], TO-DAG [11], CAPRI [12]). The structure learning algorithm typically consists of two steps. The first step involves constraining the search space of valid solutions by using statistical tests or causal theories [13]. The second step involves fitting the model to the data by maximizing the likelihood of the model and using regularization to prevent overfitting [14–16]. The third type of model is capable of inferring a general directed graph without imposing any structure constraints (e.g., NAM [17], Mutual Hazard Networks [18]). These methods use the oncogenetic graph to parameterize the transition probability matrix of a continuous-time Markov chain, which models the accumulation of events over time.

The main limitation of existing oncogenetic models is that they do not explicitly include time variables in the algorithm design, as the progression time of a sample is often unknown. However, temporal information is crucial in the oncogenetic analysis as it can reflect the trends and patterns of the event accumulation process in cancer development, potentially enhancing the reliability of oncogenetic models. To estimate the temporal order of samples, researchers have developed numerous trajectory analysis methods [19] that estimate the pseudo-time of a sample by measuring its distance along the progression trajectory. These methods have been used to infer progression roadmaps and identify drivers and regulators involved in the development of breast and bladder cancer [20, 21]. However, to the best of our knowledge, no existing method utilizes the progression time of samples to learn the oncogenetic graph.

This paper introduces TimedHN, a novel statistical model that incorporates temporal information to improve oncogenetic modeling accuracy. TimedHN has the capability to take pseudo-times as fixed input to infer the oncogenetic graph, or jointly infer the times and oncogenetic graph without pseudo-time. This feature enables TimedHN to be applied to datasets without established progression roadmaps, making it more versatile than existing methods. TimedHN models progression as a continuous-time Markov chain parameterized by a hazard network, following the Mutual Hazard Networks approach [18]. In contrast to previous models, TimedHN includes times as observable variables in the objective function, rather than marginalizing them. The hazard network and progression times of samples are estimated by solving a constrained maximum likelihood problem using the backpropagation algorithm [22]. To handle the long-tailed nature of mutation profiles [23, 24], an efficient method is developed to compute the likelihood and its gradient in a subspace of all states. This method significantly reduces the model’s memory and time complexity. Finally, TimedHN can compute the maximum likelihood transition path and the expectation of progression time for each sample using the estimated hazard network, allowing it to estimate both the temporal order of events and the temporal order of samples.

To evaluate the performance of the proposed method, we conducted several experiments. Firstly, we used synthetic data to compare the precision, recall, and F-score of TimedHN against three state-of-the-art methods and classic oncotrees. Secondly, we compared TimedHN against itself using actual sampling times as constants, demonstrating the accuracy and effectiveness of our joint inference algorithm. Thirdly, we tested the robustness of our model to profile errors by conducting experiments using noisy data. Our simulations demonstrated that the time cost of our gradient computation algorithm is linear to the total number of events and exponential to the number of accumulated events, which is typically much smaller than the total number of events. Finally, we applied TimedHN to a real-world luminal breast cancer dataset [25] to further demonstrate its practical applicability and performance.

## Methods

We propose a model for the event accumulation process based on a continuous-time Markov chain parameterized by a weighted directed graph. To determine the optimal parameters for the graph and progression times, we employ the backpropagation algorithm to maximize the log-likelihood, subject to certain constraints. In order to analyze cross-sectional data, we assume that the samples are independent. To efficiently compute the gradient, we develop an algorithm that avoids constructing the full transition matrix, significantly reducing computational complexity.

### Model overview

**Fig 1:**
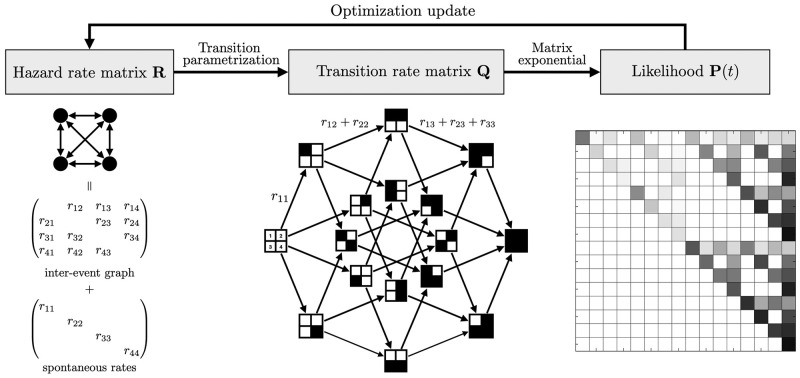
Overview of the proposed method.

Following the Mutual Hazard Networks [18], we model the mutation accumulation of *n* genetic events in cancer progression as a continuous-time Markov chain (CTMC) on 2^*n*^ states. States are represented by *n* dimensional binary vectors **x** ∈ {0, 1}^*n*^, where **x**_*i*_ = 1 means that event *i* has occurred in the tumour by time *t*, while **x**_*i*_ = 0 means that it has not. We assumed that every progression trajectory starts at a normal state **x** = (0, 0, ⋯, 0), accumulates *irreversible* genetic alteration events *one at a time*, and will eventually end at a fully aberrant state **x** = (1, 1, ⋯, 1). Observed sample profiles correspond to states at unknown intermediate times 0 < *t* < ∞ from independent progression. Fig 1 provided an overview of the method.

In a TimedHN, transition rates are parameterized by a hazard rate matrix **R**, which can be decomposed into an inter-event graph and spontaneous rates. The transition rate matrix **Q** is constrained to have a hypercube structure due to the accumulation assumptions. The transition probability matrix **P**(*t*) is computed using the matrix exponential of **Q**, which reflects the probability of transitioning from one state to another after a time interval *t*. Therefore, sample likelihood at a given time is the probability of transitioning from the normal state to the corresponding state.

### Hazard network

We use a weighted directed graph (hazard network) with adjacency matrix $R\in R^{{+}^{n\times n}}$ to represent the pairwise dependencies and use the weights to parameterize the transition rate matrix describing the accumulation process. Specifically, the model was built with three assumptions: First, for any event *j*, its waiting time without being affected by other events has an exponential distribution *t*_*i*_|**0** ∼ Exp(*R*_*ii*_). We call it a spontaneous accumulation, and *R*_*ii*_ is the spontaneous rate. Second, without considering the spontaneous accumulation, the waiting time of event *j* under the influence of event *i* also has an exponential distribution, *t*_*j*_|*i* ∼ Exp(*R*_*ij*_). Third, we assumed that the pairwise dependencies between events are independent. Then, we can use these rates to model the conditional waiting time for any event, for example, for a state **x** = (⋯, *x*_*j*−1_, 0, *x*_*j*+ 1_, ⋯) to acquire the event *j*, the conditional waiting time is the minimum of all the independent waiting times: *t*_*j*_|**x** = min(*t*_*j*_|**0**, *t*_*j*_|*m*_1_, …, *t*_*j*_|*m*_*k*_), where *k* is the number of accumulated events and *m*_*j*_ is the index of the *j*-th happened event in state **x**. By the property of competing exponentials [26], *t*_*j*_|**x** is also exponentially distributed. Specifically, the distribution of the conditional waiting time is:

$$\begin{array}{c} t_{j}|x∼\text{Exp}\left(R_{jj}+\sum_{i=1}^{n}R_{ij}x_{i}\right). \end{array}$$ (1)

### Transition rate matrix

Next, we show that the event accumulation process is equivalent to a continuous-time Markov process on 2^*n*^ states that is uniquely defined by a transition rate matrix $Q\in R^{{+}^{2^{n}\times 2^{n}}}$. The states are ordered by a index function dec(**x**) = **x**⋅**b** + 1, where **b** = (2^0^, ⋯, 2^*n*−1^)^*T*^ is the basis vector. Due to the progression assumptions: events are *irreversible* and accumulated *one at a time*, the transition can only happen between two states that differ by one entry. For example, from state **x** = (⋯, *x*_*j*−1_, 0, *x*_*j*+ 1_, ⋯) to state **x**_ + *j*_ = (⋯, *x*_*j*−1_, 1, *x*_*j*+ 1_, ⋯). The diagonal entries are defined as: *Q*_*j*, *j*_ = −∑_*i* ≠ dec(**x**_ + *j*_)_*Q*_*i*,dec(**x**_ + *j*_)_ so that rows sum to zero, which is required for *Q* to be a valid transition rate matrix of a CTMC. The transition rate from state **x** to **x**_+*j*_ is defined as:

$$\begin{array}{c} Q_{\text{dec}\left(x\right),\text{dec}\left(x_{+j}\right)}=\underset{\Delta t\to 0}{\text{lim}}\frac{P(X\left(t+\Delta t\right)=x_{+j}|X\left(t\right)=x)}{\Delta t}, \end{array}$$ (2)

which by the definition of exponential distribution equals to the rate parameter of the waiting time in Eq (1):

$$\begin{array}{c} Q_{\text{dec}\left(x\right),\text{dec}\left(x_{+j}\right)}=R_{j,j}+\sum_{i=1}^{n}R_{i,j}x_{i}. \end{array}$$ (3)

### Computation in subspace

The major problem of the computation in TimedHN is the exponentially increasing number of states, which results in unfeasible computational costs of the transition probability matrix **P**(**x**) = (*e*^*t* **Q**^). We developed an efficient method using the sparsity of **x** to compute the likelihood and its gradient. Specifically, the transition matrix **Q** is transformed by a column permutation matrix **U** such that **U**^⊤^**Q** **U** keeps the upper triangular structure. The dec(**x**)-th column is mapped to the column with the smallest possible column number. For a sample that accumulated *k* events, in the {*m*_1_, *m*_2_, ⋯, *m*_*k*_} entries, the smallest possible column number is 2^k^. We can write the column permutation as 2^k^ independent transpositions that swap the *i*-th column and the (bit_*k*_(*i* − 1)⋅**b**_sub_ + 1)-th column, for *i* = 1, ⋯, 2^*k*^, where $b_{\text{sub}}={\left(2^{m_{1}},2^{m_{2}},\cdots ,2^{m_{k}}\right)}^{T}$ is the subspace basis vector and bit_*k*_(⋅) is the inverse function of dec(⋅) that map a integer to its *k* dimension binary vector. Thus, due to the upper triangular property [27, 28] (S1 Appendix), we can get the likelihood by computing only the matrix exponential of the 2^k^-th order leading principal submatrix $\tilde{Q}=U^{⊤}{\text{QU}}_{1:2^{k},1:2^{k}}$ as:

$$\begin{array}{c} P\left(x,t\right)={\left(e^{tQ}\right)}_{1,\text{dec}(x)}={\left(e^{t\tilde{Q}}\right)}_{1,2^{k}}. \end{array}$$ (4)

And the conditional time expectation is given by:

$$\begin{array}{c} E\left(t|x\right)=\int_{0}^{\infty }t\cdot \frac{{\left(e^{tQ}\right)}_{1,\text{dec}(x)}}{\int_{0}^{\infty }{\left(e^{tQ}\right)}_{1,\text{dec}(x)}dt}dt=\frac{{\left({\left({\tilde{Q}}^{2}\right)}^{-1}\right)}_{1,2^{k}}}{-{\left({\tilde{Q}}^{-1}\right)}_{1,2^{k}}} \end{array}$$ (5)

### Optimization objective

Next, we propose to infer the hazard network through constrained maximum likelihood estimation. The goal of this approach is to identify the set of hazard rates that best explain the observed data while also incorporating prior knowledge about the progression provided by pseudo-time. The objective function is given as follows:

$$\begin{array}{cc} \underset{R,t}{\text{maximize}} & \frac{1}{\left|D\right|}\sum_{i=1}^{\left|D\right|}\text{log}\;\underset{\text{sample}\;\text{likelihood}}{\underset{︸}{\left({\left(e^{t_{i}Q}\right)}_{1,\text{dec}(x_{i})}\right)}}-\lambda \cdot |R|\\ \text{subject}\;\text{to} & R,t\geq {0,||t||}_{1}=c. \end{array}$$ (6)

The likelihood is given by *P*(**x**, *t*) = (*e*^*t* **Q**^)_1, dec(**x**)_, where *t* may be fixed to pre-defined pseudo-time values if they are available, or treated as trainable parameters otherwise. The function dec(**x**) maps the state **x** to its corresponding column index in the transition matrix. To ensure the validity of the model, we impose three constraints: (1) the hazard rates must be non-negative, as they are parameters of the exponential distribution; (2) we use ℓ1 regularization to encourage the sparseness of the matrix **R**, which results in a simpler topology of the hazard network and helps prevent overfitting; and (3) the observation times of all states must be non-negative and have a constant summation. This last constraint helps to prevent the hazard rates from becoming too small, which can occur due to the regularization term. By bounding the times, we can ensure the hazard rates do not vanish. In our implementation, we use the ReLU (rectified linear unit) activation function to ensure that all hazard rates in the **R** matrix are positive. We scale times after each gradient step to maintain a constant summation.

### Backpropagation in the subspace

To optimize the objective function using the backpropagation algorithm, we need to calculate the partial derivatives of the likelihood with respect to hazard rate matrix **R** and time *t*. These partial derivatives are given as:

$$\begin{array}{cc} \frac{\partial {\left(e^{tQ}\right)}_{1,\text{dec}(x)}}{\partial R} & =\sum_{i,j}\frac{\partial {\left(e^{tQ}\right)}_{1,\text{dec}(x)}}{\partial Q_{i,j}}\frac{\partial Q_{i,j}}{\partial R}. \end{array}$$ (7)

$$\begin{array}{cc} \frac{\partial {\left(e^{tQ}\right)}_{1,\text{dec}(x)}}{\partial t} & ={\left(Qe^{tQ}\right)}_{1,\text{dec}(x)}. \end{array}$$ (8)

Since the likelihood equals to ${\left(e^{t\tilde{Q}}\right)}_{1,2^{k}}$ only depends on $\tilde{Q}$, the derivatives could be computed efficiently in the subspace as:

$$\begin{array}{cc} \frac{\partial {\left(e^{tQ}\right)}_{1,\text{dec}(x)}}{\partial Q} & =U(\begin{array}{cc} \partial {\left(e^{t\tilde{Q}}\right)}_{1,2^{k}}/\partial \tilde{Q} & 0\\ 0 & 0 \end{array})U^{⊤} \end{array}$$ (9)

$$\begin{array}{cc} \frac{\partial {\left(e^{tQ}\right)}_{1,\text{dec}(x)}}{\partial t} & ={\left(\tilde{Q}e^{t\tilde{Q}}\right)}_{1,2^{k}}. \end{array}$$ (10)

As shown in the computation in S1 Appendix the derivative of matrix exponential required in Eq (9) is given as: $\partial {\left(e^{t\tilde{Q}}\right)}_{1,2^{k}}/\partial \tilde{Q}={\left(te^{B}\right)}_{1:2^{k},2^{k}+1:2^{k+1}}$, where matrix **B** is constructed as:

$$\begin{array}{c} B=(\begin{array}{cc} t{\tilde{Q}}^{⊤} & E_{1,2^{k}}\\ 0 & t{\tilde{Q}}^{⊤} \end{array}). \end{array}$$ (11)

## Results

### Simulation on synthetic datasets

We sample synthetic data using CTMCs parameterized by random hazard networks to test the performance of TimedHN in inferring the structure of hazard networks using a given amount of data. We set the numbers of events to *n* = 15 and tested different sample sizes |D|∈{100,250,500,1000}. We used hazard networks with forest and directed acrylic graph (DAG) structure to parameterize CTMCs. For each combination of sample size |D| and topology type, we generated 100 sets of data using different randomly generated hazard networks.

#### Generation of random hazard networks

To generate forests, we set a maximum depth of *log*(*n*) and assign each node a random depth between 1 and ⎿*log*(*n*)⏌, ensuring that each depth has at least one node. We then randomly select a parent from the nodes in the previous depth for each node. To generate DAGs, we first assign topological sort ranks to the event nodes. We then randomly connect a higher-ranked node to a lower-ranked node to form ⎿1.5*n*⏌ inter-event edges. For simplicity, we set all edge weights to 1. In addition, the spontaneous rates of all the source nodes are set to 1, and the spontaneous rates of all the rest nodes are set to 0.1.

#### Event profiles sampling

In real-world data, we observed that most samples accumulate fewer than 10 mutations in cancer-related genes. This observation may be because accumulating more mutations could make a cell less viable, therefore, less frequently observed. To reflect this phenomenon in our simulation, we set the maximum number of accumulated events to 10. Specifically, we let a CTMC transition ten times to get one simulation run of the accumulation process. We then use the time *T* of the tenth jump as the termination time. Finally, we randomly sample an observation time *t* ∈ [0, *T*] and use the state of the CTMC at time *t* as a data sample. This sampling process is repeated multiple times to generate independent samples of synthetic datasets.

#### Performance measure

Algorithmic performance was evaluated using the metrics precision, recall, and F-score between the inferred and true graphs used in the simulation. Precision and recall are defined as follows: precision tp/(tp+fp), recall tp/(tp + fn), and F-score 2tp/(2tp + fp + fn) which is the harmonic mean of precision and recall, where tp are the true positives, fp are the false positives and fn are the false negatives. Values for precision, recall, and F-score range from 0 to 1. The closer to 1, the better.

#### Experimental settings

We compared our method with Mutual Hazard Networks (MHN) [8, 18], CAPRESE [7], CAPRI [12], and oncotrees [4]. For MHN, we used the source code downloaded from the paper websites. The L1 constraint weight for MHN is set to 1/|D| as suggested in the original paper. We used the implementation in the R package TRONCO (2.26.0) [29] with the default parameter settings for CAPRESE and CAPRI. For oncotrees, we used our python implementation. For TimedHN, we used the proposed method to maximize the average log-likelihood in Eq (6) and set the learning rate to 1*e* − 3. We found that a larger regularization parameter *λ* usually results in more false negatives, which results in a low recall. On the other hand, although a smaller regularization parameter results in more false positives, the weights of true positive edges are usually much larger. Thus we can effectively remove false positive edges by using a threshold. In the simulation experiment, we used *λ* = 1*e* − 2, and we used 0.1max(**R**) as the threshold. However, the threshold is a hyperparameter and could be set manually after the optimization based on the user’s preference over precision and recall.

#### Benchmark experiment on synthetic datasets

**Fig 2:**
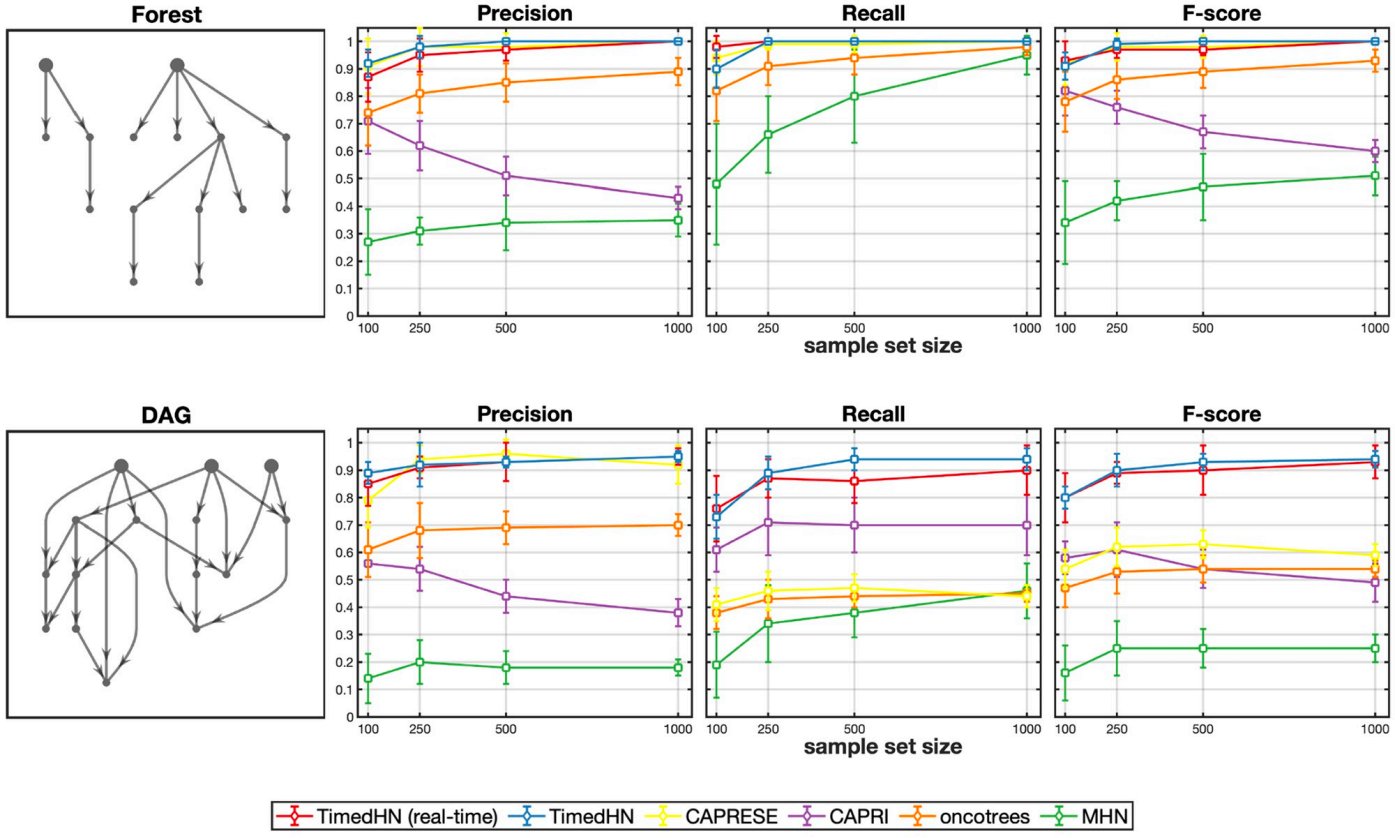
The precision, recall, and F-score of the six methods was compared on synthetic datasets consisting of 15 events sampled from CTMCs parameterized by forests and DAGs.

We compared the performance of TimedHN and four competing methods for inferring trees and DAGs using synthetic data. We also tested the TimedHN with true time observations instead of inferring times to demonstrate the advantage of the joint inference algorithm. Fig 2 showed the performance of the six methods on simulations of 15 events with different sample sizes (|D|∈{100,250,500,1000}), obtained by averaging over 100 runs.

We apply six methods to infer trees and forests, where each event has only one parent event. CAPRESE and TimedHN use real-time and joint inference, performing almost perfectly when sample sizes are larger than 500. Since CAPRESE is designed only to infer a tree or forest structure, this simulation perfectly fits its assumptions. Although oncotrees’ simple heuristic does not lead to perfect performance, it assumes a tree structure. Thus it still significantly outperforms CAPRI and MHN, which do not assume a tree structure. On the contrary, TimedHN converges to the correct structure without these assumptions. Our results showed that the precision and F-score of CAPRI decrease as the sample size increases. This is because CAPRI tends to infer a denser graph on larger sample sets, resulting in a higher false positive rate and recall. We find MHN performs poorly even in this simple case. Two possible reasons are (i) it assumes an identical distribution for progression times *P*(*t*) for all samples, while the conditional distributions *P*(*t*|**x**) are different, which could lead to an erroneous topology of the hazard network. (ii) MHN also tries to infer negative hazard rates, which means the searching space of its optimization algorithm is much more complicated. Thus, it is easier to converge to a local optimum or result in overfitting. Moreover, in section, we find that MHN prefers to use edges with negative weights to fit the data rather than adding edges with positive weights.

Then, we apply the six methods to infer DAGs, where events can have multiple parent events. In terms of precision, CAPRESE is still comparable with TimedHN. However, in terms of recall, TimedHN using real-time or joint inference outperforms all competing methods. TimedHN using joint inference performs slightly better than the actual time and has a smaller standard deviation. A possible explanation is that joint inference reduces the variance of sampling times. Due to the tree and forest assumption, oncotrees and CAPRESE can infer (*n* − 1) edges at most. Thus their recall is low when there are 1.5*n* edges in the true hazard networks. The recall of CAPRI decreases dramatically compared to their results on forests. Because its *prima facie causality* rules could fail in this case since the probability of observing a parent event is not guaranteed to be larger than that of observing a child. Finally, TimedHN significantly outperforms all competing methods in F-score due to its good performance on precision and recall.

#### Experiment with profile noise

**Fig 3:**
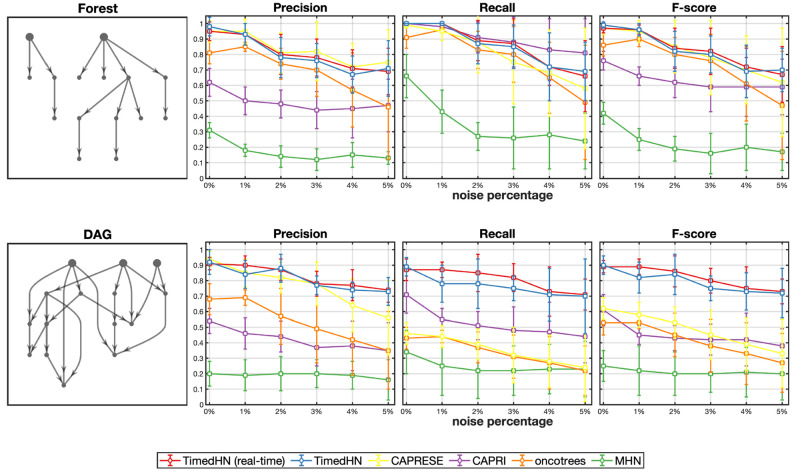
The precision, recall, and F-score of six methods was compared on synthetic datasets with 15 events. and uniform noise at six noise level (0.1%, 0.25%, 0.5%, 1%, 2.5%, 5%). The datasets were generated by sampling from CTMCs parameterized by forests and DAGs. Error bars represent one standard deviation.

We conducted simulation experiments to evaluate the robustness of our model to observation errors and compared the results with four competing methods. We used datasets of size |D|=250 generated from forest and DAG structures with *n* = 15 events. We randomly generated 100 datasets for each topology type using different hazard networks and added noise by flipping each event independently with a small probability. Fig 3 shows the performance of the six methods. As expected, the performance of all methods decreased as the noise level increased. However, TimedHN using real-time and joint inference remained comparable to CAPRESE in inferring forests and outperformed all competing methods in inferring DAGs at all noise levels. For forest structure, CAPRESE and Oncotrees performed better than CAPRI and MHN due to their structure assumptions. We found that TimedHN sometimes had to infer false edges to maintain positive likelihoods for defected profiles. However, the weights of these false edges were usually small enough to be removed by a threshold when the number of errors was small. When the number of profile errors was large, the false positive edges became indistinguishable from edges connecting low-frequency events.

### Time complexity analysis

**Fig 4:**
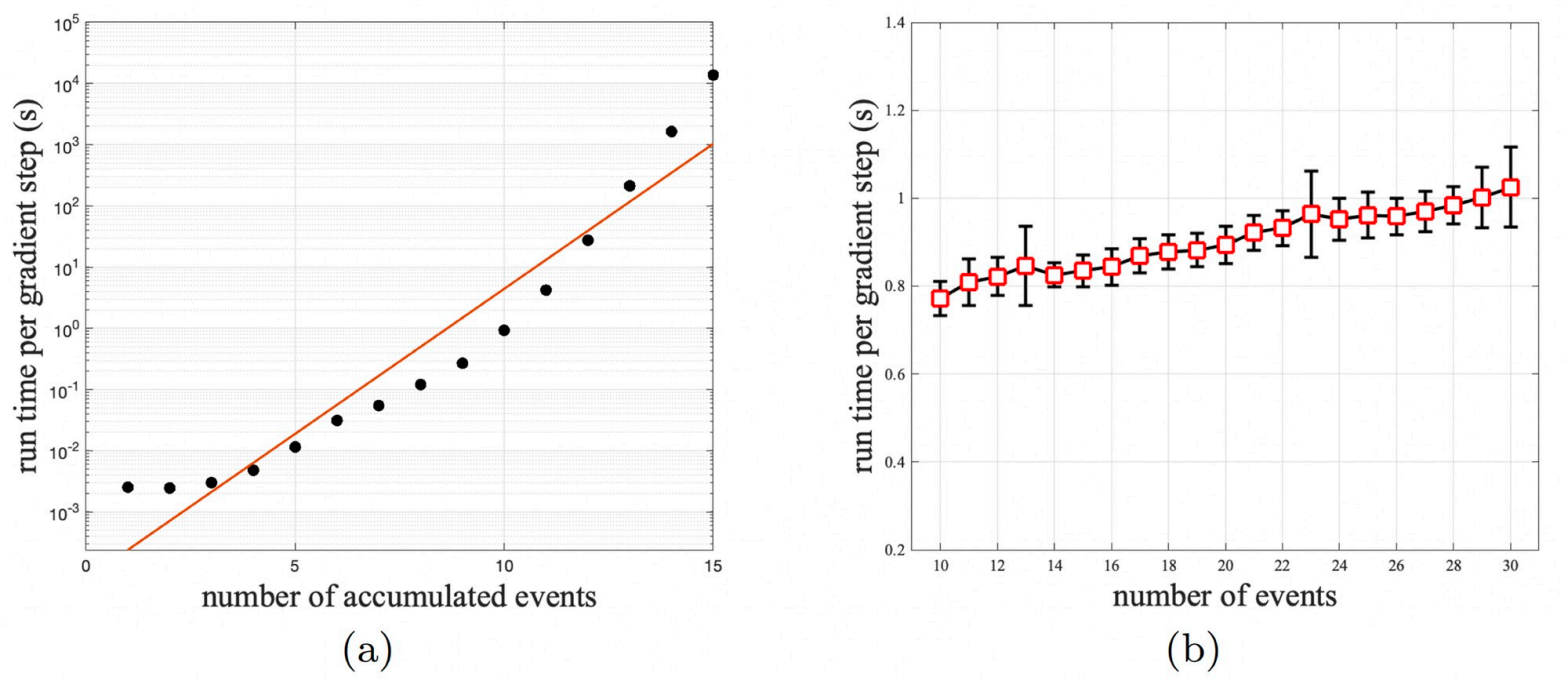
The time cost of one gradient step is plot against different (a) number of accumulated events *k* and (b) number of events *n*.

We performed a test to analyze the time complexity of our gradient computation algorithm. We first fixed the profile dimension *n* = 20 and tested different numbers of accumulated events *k* ∈ {1, ⋯, 15}. In Fig 4(a), we can see the run time of gradient computation increased exponentially to *k*. It is because the computation of matrix exponential is the most time-consuming step, and the size of the matrix $\tilde{Q}$ in section grows exponentially to *k*.

Then, we fix the number of accumulated events to *k* = 10 and test different profile dimensions *n* ∈ 10, 11, …, 30. Fig 4(b) shows that the computation time increases slowly as *n* increases. This is because since *k* is fixed, only the size of the permutation matrix **U** is increasing linearly to *n* using sparse representation. These results show that our algorithm can take advantage of the sparsity of event profiles. When most patients just accumulate less than 10 cancer-related events, our algorithm can compute the gradient efficiently, regardless of the total number of events.

### Luminal breast cancer

In this study, we compared the performance of TimedHN to CAPRESE and MHN using luminal breast cancer data from The Cancer Genome Atlas (TCGA) [25, 30]. The dataset, which consists of 685 profiles of luminal A and luminal B subtypes, was previously used in the CancerMapp pipeline [20]. We obtained event profiles from the Mutation Annotation Format (MAF) file used for the MutSig2CV [31] mutation analysis in TCGA, which catalogs mutations in 15,889 genes in 973 breast tumor samples. These mutations were classified into five categories: missense, nonsense, in-frame indels, frameshift indels, and splice site. As these types of mutations can damage the function of a gene to varying degrees by altering the amino acid sequence or disrupting the translation process, we treated all of them as non-silent mutations of a gene in our analysis.

To select genes for our experiment, we used the CancerMapp pipeline [20], which applies a statistical approach to identify significant changes in gene mutations along a progression model inferred from expression profiles. We applied the Benjamini-Hochberg procedure [32] to compute a false discovery rate (FDR) for each gene, and only included those with an FDR lower than 0.01 in our analysis (see S1 Table).

**Fig 5:**
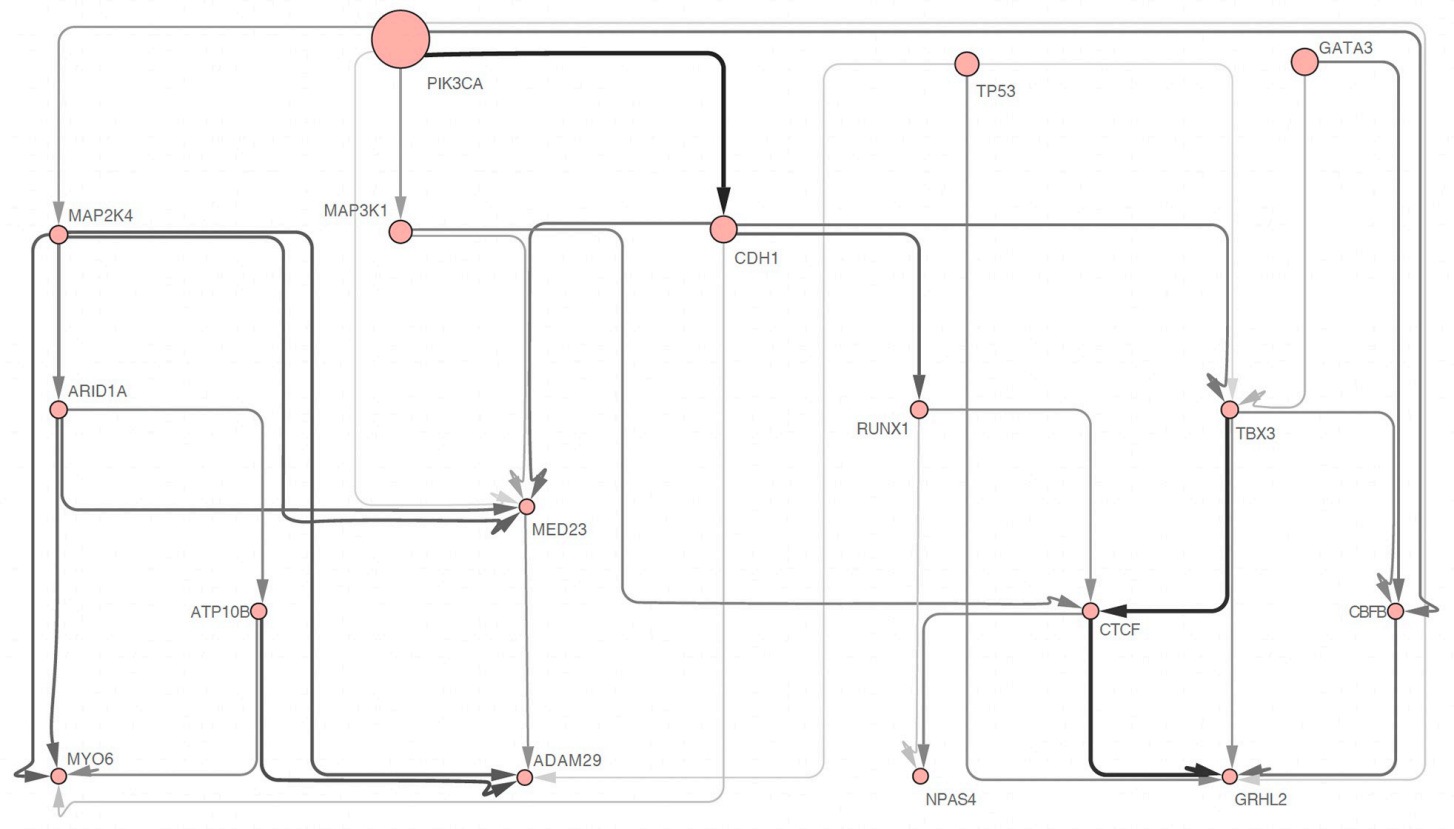
The oncogenetic graph inferred by TimedHN. Edge widths and shade are linear to the inter-event hazard rates. The node sizes are linear to spontaneous hazard rates.

**Fig 6:**
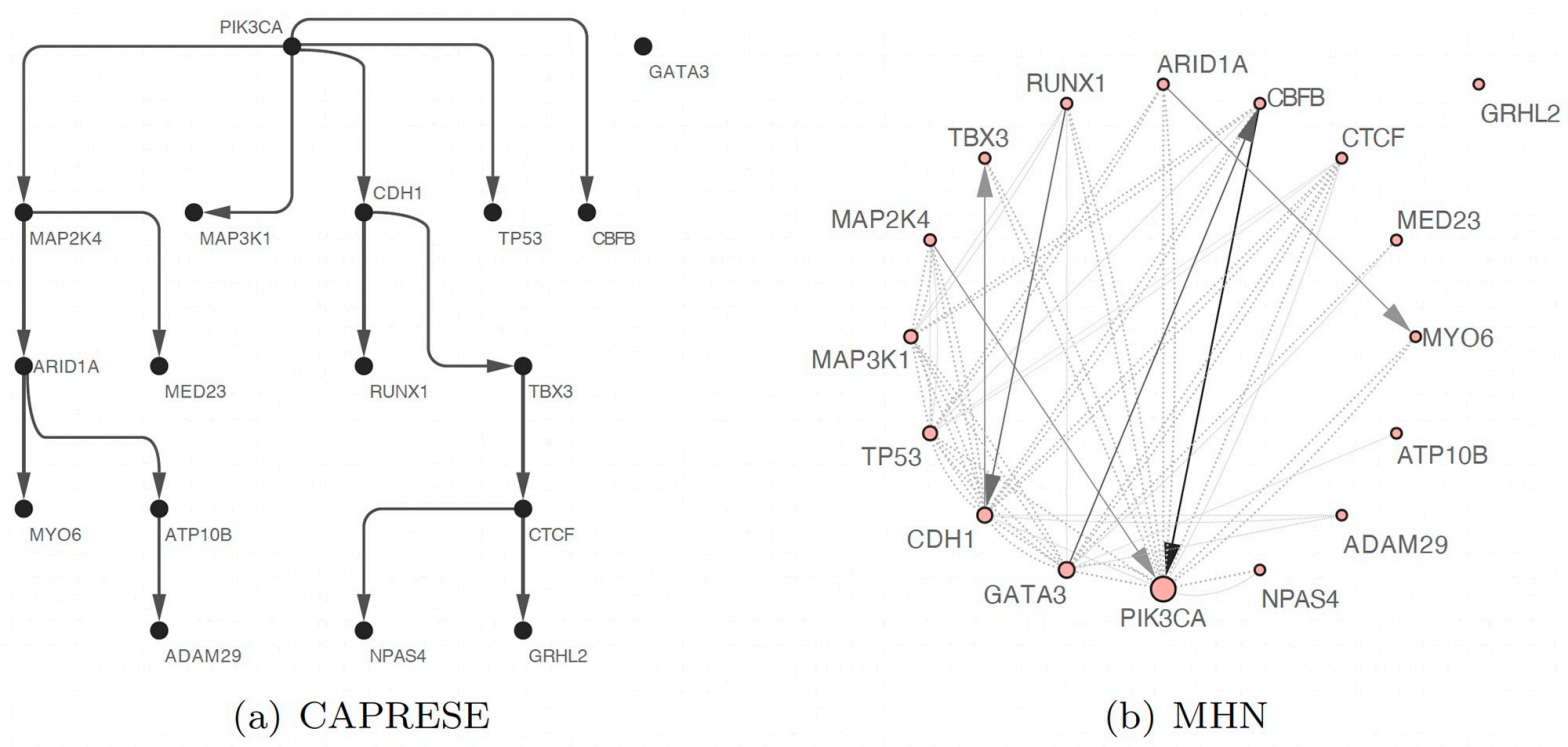
The oncogenetic tree inferred by CAPRESE is shown in subfigure (a) Its edges and nodes are shown in uniform width and size. The Oncogenetic graph inferred by MHN is shown in subfigure (b). Its edge widths and shade are linear to the exponential of inter-event hazard rates. Node sizes are linear to the exponential of spontaneous hazard rates. Solid lines represent edges with positive weights, and dashed lines represent negative ones.

We compared the results of TimedHN to CAPRESE, which demonstrated the highest precision level in the benchmark experiment. As shown in Fig 5, TimedHN was able to capture all of the edges identified by CAPRESE shown in Fig 6(a), except for the edge from PIK3CA to TP53. Instead, TimedHN identified TP53 as a source node with a high spontaneous rate to fit the 36 samples in the dataset that had TP53 mutations but not PIK3CA mutations. The independence of PIK3CA and TP53 inferred by TimedHN is consistent with their known roles as an oncogene and tumor suppressor, respectively, suggesting that abnormalities in either gene can promote a malignant phenotype [33].

TimedHN has inferred several edges that are consistent with the findings of other research studies. For example, the predicted interaction between PIK3CA and MAP3K1/MAP2K4 mutations is in line with the fact that these genes cooperate at both the mutation and pathway levels [34]. The inferred edge from PIK3CA to CDH1 is consistent with the finding in lung adenocarcinoma that the inactivation of the PI3K pathway significantly reduced CDH1 expression [35]. The predicted interaction between CDH1 and RUNX1 is consistent with the observation of significant enrichment of RUNX1 binding on E-cadherin (CDH1) in breast cancer cells [36]. The connection between RUNX1 and CTCF is consistent with the findings that CTCF suppresses RUNX1 expression [37], thus the loss of CTCF will further cause an over-expression of RUNX1, which can lead to the proliferation of abnormal cells.

However, TimedHN identified several edges that were not inferred by CAPRESE or reported in previous research. These include the GATA3, CBFB, GRHL2 series, the confluence of MAP3K1, MAP2K4, and CDH1 to MED23, and the edges connecting MAP3K1 to CTCF, MAP2K4 to ADAM29, and MAP2K4 to MYO6. While some of these edges may not be as significant as those identified by CAPRESE, they are strong enough to withstand the ℓ_1_ norm regularization and thresholding. These findings suggest that these edges reflect valuable patterns in the dataset, although further studies are needed to confirm this hypothesis.

We also compared the results of Mutual Hazard Networks (MHN, Fig 6(b)) to demonstrate its limitations. MHN inferred many mutually exclusive relationships using a fully connected subgraph with only negative weighted edges, such as the mutual exclusiveness between MAP3K1, MAP2K4, and CDH1. However, this approach is expensive in terms of ℓ_1_ cost, and as a result, the model tends to trade positive edges to model such a dense subgraph. Additionally, due to the incorrect assumption of the conditional time distribution *P*(*t*|**x**), the direction of some inferred edges with positive weight is reversed compared to the results from TimedHN and CAPRESE. In contrast, methods like CAPRESE and TimedHN that only infer positive dependencies can also represent mutually exclusive relationships by disconnection. Allowing negative hazard rates would significantly expand and complicate the search space in optimization, which could lead to convergence to a local optimum or overfitting.

Finally, as shown in S2 Table, TimedHN demonstrated the ability to estimate the pseudo-time order of profiles and events. Event profiles were sorted by the conditional time expectation, and a brute force search of all possible accumulation orders of events in a profile was used to find the maximum likelihood accumulation order.

## Conclusion

In this study, we present TimedHN, a new framework for inferring the temporal order of samples and the oncogenetic graph underlying the accumulation of genetic events in cancer progression. We developed an efficient gradient computation algorithm that can take advantage of data sparsity and significantly reduce the computational complexity of the proposed model. In our experiments on synthetic datasets, we proved the correctness and robustness of TimedHN by showing convergence to the correct typology. We compared TimedHN to the state-of-the-art tree reconstruction algorithm (CAPRESE), bayesian probabilistic graphical model (CAPRI), and Mutual Hazard Networks (MHN). The results showed that TimedHN outperforms them on synthetic data. Furthermore, we experimented on luminal breast cancer mutation data using CAPRESE, MHN, and TimedHN. The analysis suggested that the results of TimedHN are highly consistent with the most precise method, CAPRESE, in the simulation and can infer novel dependencies that are undetected by CAPRESE. At the same time, the analysis of the result of MHN showed its limitation in reliability and ease of interpretation.

Despite its strengths, TimedHN has some limitations that should be acknowledged. One limitation is that its application is highly dependent on the selection of genetic events, as it is only able to infer meaningful results on a pre-selected set of events that are thought to be involved in a cumulative causal process. This is a limitation shared by all oncogenetic graph learning methods. TimedHN can still be useful as a tool for providing computational evidence for such hypotheses or for identifying potential oncogenetic dependencies. Another limitation is that the computational complexity of the proposed algorithm is still exponential in the number of accumulated events, making it only efficient for sparse profiles.

There are several directions for future research that could expand the capabilities and applicability of TimedHN. One possibility is to improve the scalability and efficiency of the tool, such as through approximations or alternative optimization techniques. This would make it more widely applicable and useful for researchers, particularly in cases where data is not sparse. Another potential direction is to apply TimedHN to multi-region and single-cell data, as the framework is capable of computing the transition probability between any two states. This would allow researchers to utilize datasets from different sources and benefit from the results of phylogenetic analysis [38–40]. Additionally, TimedHN could be applied to the analysis of other disease progressions or cumulative causal processes, such as the development of drug resistance-associated mutations in the HIV genome [41] or similar processes. This would allow researchers to leverage the strengths of TimedHN in a wider range of research contexts.

We expect that in the future, TimedHN will be a valuable resource for cancer research and provide new insights into the development of more effective targeted therapies.

## Supporting information

S1 Appendix Gradient computation of matrix exponential. A detialed derivation of the gradient of the likelihood function (matrix exponential).
(PDF)

S1 Table Driver genes for luminal breast cancer. Genes selected by CancerMapp pipeline that showed significant changes along the progression of gene expression profiles.
(CSV)

S2 Table Pseudo-time analysis for mutations and patients. Maximum likelihood estimation of the accumulation orders and conditional expectation of progression times for all unique profiles.
(XLSX)
