# Supplementary material for: Timed hazard networks: Incorporating temporal difference for oncogenetic analysis
Source: PLoS One. 2023 Mar 16;18(3):e0283004. doi: 10.1371/journal.pone.0283004 (PMC10019724; doi:10.1371/journal.pone.0283004)
Supplement: S1 Appendix — A detialed derivation of the gradient of the likelihood function (matrix exponential). (PDF) [file pone.0283004.s001.pdf]

S1 Appendix. Gradient computation of matrix exponential A detialed derivation of the gradient of the likelihood function (matrix exponential)

Matrix exponential for block upper triangular matrix

If a square matrix $\mathbf{A}$ is block upper triangular [1, 2],

$$\mathbf{A} = \begin{pmatrix} \mathbf{A}_{11} & \mathbf{A}_{12} \\ 0 & \mathbf{A}_{22} \end{pmatrix}, \tag{1}$$

where $\mathbf{A}_{11}$ and $\mathbf{A}_{22}$ are square matrices. The matrix exponential has the same structure.

$$e^{\mathbf{A}} = \begin{pmatrix} e^{\mathbf{A}_{11}} & \mathbf{F} \\ 0 & e^{\mathbf{A}_{22}} \end{pmatrix}. \tag{2}$$

Where:

$$F = \int_0^1 e^{(1-u)\mathbf{A}_{11}} \mathbf{A}_{12} e^{u\mathbf{A}_{22}} du. \tag{3}$$

Derivatives of matrix exponential

For an order n square matrix $t\mathbf{A}$, the partial derivative of the (i, j)-th entry of its matrix exponential $e^A \in \mathbb{R}^{+n\times n}$ with respect to $\mathbf{A}$ is defined as:

$$\frac{\partial (e^{t\mathbf{A}})_{ij}}{\partial \mathbf{A}} = \begin{pmatrix} \partial (e^{t\mathbf{A}})_{ij}/\partial A_{11} & \cdots & \partial (e^{t\mathbf{A}})_{ij}/\partial A_{1n} \\ \vdots & \ddots & \vdots \\ \partial (e^{t\mathbf{A}})_{ij}/\partial A_{n1} & \cdots & \partial (e^{t\mathbf{A}})_{ij}/\partial A_{nn} \end{pmatrix}, \tag{4}$$

which is related to the first directional direvative (Gateaux derivative) of matrix exponential:

$$\frac{\partial (e^{t\mathbf{A}})}{\partial A_{ij}} = \lim_{h\to 0} \frac{1}{h} \left(e^{t(\mathbf{A}+h\mathbf{E}_{i,j})} - e^{t\mathbf{A}} \right), \tag{5}$$

$$= \int_0^t e^{(t-\tau)\mathbf{A}} \mathbf{E}_{i,j} e^{\tau\mathbf{A}} d\tau, \tag{6}$$

where $\mathbf{E}_{i,j}$ is a direction matrix, it has only one non-zero value at the (i, j)-th entry which equals to 1. According to the *proposition* 6.1 in [3],

$$\frac{\partial (e^{\mathbf{A}})_{ij}}{\partial \mathbf{A}} = \frac{\partial e^{\mathbf{A}^\top}}{\partial A_{ij}}. \tag{7}$$

Thus, we have the closed form solution for Eq.(4):

$$\frac{\partial (e^{t\mathbf{A}})_{i,j}}{\partial \mathbf{A}} = \int_0^t e^{(t-\tau)\mathbf{A}^\top} \mathbf{E}_{i,j} e^{\tau\mathbf{A}^\top} d\tau. \tag{8}$$

Since the numerical computation of the integral in Eq. (8) is time comsuming, we give an analytic solution for it.

$$\frac{\partial (e^{t\mathbf{A}})_{i,j}}{\partial \mathbf{A}} = \int_0^t e^{(t-\tau)\mathbf{A}^\top} \mathbf{E}_{i,j} e^{\tau\mathbf{A}^\top} d\tau, \tag{9}$$

$$= \int_0^1 e^{(t-tu)\mathbf{A}^\top} \mathbf{E}_{i,j} e^{tu\mathbf{A}^\top} d(tu), \tag{10}$$

$$= t \int_0^1 e^{(1-u)(t\mathbf{A}^\top)} \mathbf{E}_{i,j} e^{u(t\mathbf{A}^\top)} du. \tag{11}$$

Following Eq. (3) and Eq. (11), we can then construct an order $2n$ square matrix $\mathbf{B}$:

$$\mathbf{B} = \begin{pmatrix} t\mathbf{A}^\top & \mathbf{E}_{i,j} \\ 0 & t\mathbf{A}^\top \end{pmatrix}, \tag{12}$$

And compute the derivative as:

$$te^{\mathbf{B}} = \begin{pmatrix} te^{t\mathbf{A}^\top} & \partial(e^{t\mathbf{A}})_{ij}/\partial\mathbf{A} \\ 0 & te^{t\mathbf{A}^\top} \end{pmatrix}. \tag{13}$$

References

[1] Van Loan C. The Sensitivity of the Matrix Exponential. SIAM Journal on Numerical Analysis. 1977;14(6):971–981.

[2] Dieci L, Papini A. Padé approximation for the exponential of a block triangular matrix. Linear Algebra and its Applications. 2000;308(1-3):183–202.

[3] Najfeld I, Havel TF. Derivatives of the matrix exponential and their computation. Advances in Applied Mathematics. 1995;16(3):321–375.
